# Effect of oral antibiotics after two-stage revision for periprosthetic joint infection on subsequent antibiotic resistance within a national cohort of United States veterans

**DOI:** 10.5194/jbji-10-7-2025

**Published:** 2025-02-04

**Authors:** Judd Payne, Jesse D. Sutton, Brenna E. Blackburn, Snehal Bansod, Hannah Imlay, Emily S. Spivak, Jakrapun Pupaibool, Jeremy M. Gililland, Laura K. Certain

**Affiliations:** 1 George E. Wahlen VA Medical Center, Salt Lake City, Utah, United States of America; 2 Department of Internal Medicine, Division of Infectious Diseases, University of Utah, Salt Lake City, Utah, United States of America; 3 Department of Orthopaedics, University of Utah, Salt Lake City, Utah, United States of America

## Abstract

**Background**: Prior studies have indicated that administration of prolonged courses of oral antibiotics after Stage 2 reimplantation surgery for periprosthetic joint infection (PJI) results in a lower rate of recurrent PJI. However, there is concern that this antibiotic usage results in an increased risk of antibiotic resistance in any subsequent PJI that does occur.

**Methods**: We retrospectively reviewed patients who underwent Stage 2 reimplantation surgery for PJI within the national Veterans Affairs hospital system of the United States. We compared those who received at least 2 weeks of oral antibiotics after Stage 2 reimplantation to those who did not. The primary outcome was the proportion of organisms resistant to four classes of antibiotics (tetracyclines, fluoroquinolones, oral beta-lactams, and sulfonamides) in recurrent PJI. Secondary outcomes included recurrent PJI and death.

**Results**: Of the 605 patients who underwent Stage 2 reimplantation for PJI, 154 patients received at least 14 d of antibiotics after surgery and 451 patients did not. Bacteria causing recurrent PJI in patients who received prolonged antibiotics were more likely to be resistant to tetracyclines and trimethoprim–sulfamethoxazole but not oral beta-lactams or fluoroquinolones. There was no difference in risk of recurrent PJI or death between the two groups.

**Conclusions**: Prolonged oral antibiotic treatment after Stage 2 reimplantation increases the risk of antibiotic resistance to some antibiotics in subsequent PJI. We recommend further research to identify the best choice of antibiotic and duration after Stage 2 reimplantation, to maximize benefits while minimizing risks.

## 1 Introduction

Periprosthetic joint infection (PJI) is one of the most feared complications of hip and knee replacement, with high morbidity and mortality (Zmistowski et al., 2013). The management of PJI often involves a two-stage exchange in which the surgeon removes the infected prosthesis, the patient is treated with both local (antibiotic spacer) and systemic antibiotics for a prolonged period, and then a new prosthesis is placed after the infection is thought to be eradicated. However, even with this aggressive management a significant number of patients will suffer a recurrent PJI, often with a different organism, necessitating further surgeries and prolonged courses of antibiotics (Wichern et al., 2020; Petis et al., 2019b, a; Tan et al., 2018). Though rates differ depending on the definition of recurrence and the length of follow-up, a typical recurrence rate after two-stage exchange for PJI is about 15 % within 5–10 years (Petis et al., 2019b, a; Tan et al., 2018; Kelly et al., 2022; Yang et al., 2020). Interventions to reduce the rate of recurrent PJI in this population are therefore of much interest.

A randomized clinical trial first published in 2017, with additional results published in 2020, indicated that administering 3 months of oral antibiotics after Stage 2 reimplantation reduced the risk of recurrent PJI in patients treated with two-stage exchange (Frank et al., 2017; Yang et al., 2020). Though there were some concerns with study methodology (Manning et al., 2020), many surgeons adopted the practice of prescribing a 3-month course of antibiotics at the time of Stage 2 reimplantation for all patients treated with two-stage exchange. However, it is not clear that the full 3 months is necessary for benefit (Ryan et al., 2023), and antibiotic use can cause adverse effects and select for drug-resistant organisms. We previously demonstrated that extended courses of doxycycline after Stage 2 reimplantation surgery were associated with a higher risk of subsequent infection with doxycycline-resistant organisms (Kelly et al., 2022). Though provocative, that work was limited by its being from a single site and by most patients receiving the same antibiotic (doxycycline). It remained unclear whether other antibiotics would exert the same selective pressure.

The aim of the current study was to investigate the impact of extended courses of oral antibiotics after Stage 2 reimplantation in a broader patient cohort, treated with a variety of oral antibiotics. We sought to determine whether prolonged courses of antibiotics after Stage 2 reimplantation were associated with an increased risk for antibiotic resistance to various classes of antibiotics in any subsequent infections in the same joint. As a secondary analysis, we examined whether patients treated with prolonged courses of antibiotics were at lower risk of recurrent PJI.

## 2 Methods

This was a retrospective cohort study of all patients who underwent a Stage 2 reimplantation surgery for hip or knee PJI between 1 October 2015 and 1 June 2020, within the national Veterans Health Administration (VHA) system of the United States of America. Data were extracted from the Corporate Data Warehouse (CDW) through the VA Informatics and Computing Infrastructure (VINCI). We identified our patient population using a combination of procedure codes and PJI diagnosis codes, followed by manual chart review to confirm that the identified surgery was a Stage 2 reimplantation. Potential patients were identified using Current Procedural Terminology (CPT) codes 27134 or 27487 combined with 11982, CPT codes 27134 or 27487 or free text “spacer” in the operative report, or an *International Classification of Diseases – Tenth Revision* (ICD-10) procedure code for hip or knee spacer removal (i.e., OSP*08Z, OSP*0EZ, OSP*38Z, or OSP*48Z with * referring to codes specific to hips and knees). PJI was identified with ICD-9 (996.66) or ICD-10 (T84.5*) diagnosis codes in the 1 year before the surgery date. Patients with a spacer insertion code (CPT codes 11981 or 11983 or ICD-10-PCS codes 0SH*08Z) on the same date as removal were not included, because we assumed this reflected spacer replacement rather than Stage 2 reimplantation. Manual chart review of operative notes was performed to confirm that the patient had undergone Stage 2 reimplantation for PJI, because procedural coding alone was heterogenous across study facilities and inaccurate for identifying a second-stage revision.

Once we had our confirmed cohort of patients who underwent a Stage 2 reimplantation surgery for prior PJI, we extracted further data from the CDW. Age, sex, body mass index (BMI), American Society of Anesthesiologists (ASA) classification, smoking status, microbiology (all non-urine, non-respiratory culture results from 2 years before and 2 years after their Stage 2 reimplantation), Charlson Comorbidity Index (CCI), and selected medical comorbidities (rheumatoid arthritis, diabetes mellitus, chronic kidney disease, and hemoglobin A1c) were extracted from the CDW for all patients. We used outpatient pharmacy data to identify patients who filled a prescription for at least 2 weeks of oral antibiotics within 7 d following their Stage 2 reimplantation; we then confirmed this antibiotic data with manual chart review of discharge summaries. Any patients who received prolonged intravenous antibiotics at the time of Stage 2 reimplantation were excluded from the study, as were patients with positive culture results at the time of Stage 2 reimplantation. Patients with an initial fungal or mycobacterial PJI were likewise excluded.

Our primary outcome was the presence of resistant organisms in any subsequent infection in the same joint, comparing those who had received extended (≥2 weeks) oral antibiotics at the time of Stage 2 reimplantation to those who had not. We identified all patients who underwent repeat surgery for any reason on the same joint and conducted manual chart review to determine the following: the type of repeat surgery, the diagnosis of recurrent PJI, and the resistance pattern of any cultured pathogens. We considered patients to have a recurrent PJI if the operative note for the repeat surgery indicated concern for infection (e.g., purulence, sinus tract), cultures from the repeat surgery were positive, or the progress notes indicated that the treating physicians considered this patient to have a recurrent PJI. We specifically looked at resistance to trimethoprim–sulfamethoxazole, tetracyclines, oral beta-lactams, fluoroquinolones, and clindamycin. Culture-negative infections included those with no organisms detected or organisms isolated in small amounts (e.g., one colony, broth only) from only one culture sample. Microbiology data for the index PJI (i.e., not the recurrent PJI) was inferred from the culture data pulled from CDW for the 2 years prior to the Stage 2 reimplantation.

Secondary outcomes included recurrent PJI and time to recurrent PJI, comparing patients who had received extended oral antibiotics after Stage 2 reimplantation to those who had not. We also determined the incidence of adverse events from antibiotics among those veterans prescribed antibiotics after Stage 2 reimplantation. Antibiotic-associated adverse effects were identified by manual chart review, looking at laboratory values and clinician notes from the 3 months after Stage 2 reimplantation surgery. Any potential adverse effects documented in the medical record were recorded for analysis. Acute kidney injury (AKI) was defined as either a rise in serum creatinine of *>*0.3 mg dL^−1^ or as documentation in the notes of AKI. Other potential adverse effects were gathered mainly from note review: nausea/vomiting, rash, or diarrhea.

Outcomes were compared between patients who received oral antibiotics and those who did not using *t* tests, chi-squared tests, and Fisher's exact tests. Cox regression was used to compare time from Stage 2 reimplantation to recurrent PJI or death, both unadjusted and adjusted for prespecified covariates (age, BMI, CCI, and diabetes). Statistical analysis was performed using SAS 9.4 (Cary, NC, USA). We did our initial detailed chart review starting in July 2021, ensuring at least 1 year of follow-up from the time of Stage 2 reimplantation surgery.

## 3 Results

There were 840 unique patients with a procedure code for possible Stage 2 reimplantation, a diagnosis of PJI, and no procedure code reflecting spacer reinsertion. Of these 840 patients, 657 were confirmed to have Stage 2 reimplantation by manual review of operative notes. (The other 183 patients had surgeries that were not Stage 2 reimplantations, e.g., spacer exchange.) Of the 657, 52 were excluded based on manual chart review for the following reasons (patients could meet multiple criteria): positive cultures at the time of Stage 2 reimplantation (*n*=22), receipt of parenteral antibiotics at the time of Stage 2 reimplantation (*n*=17), or fungal or mycobacterial initial PJI (*n*=16). The final study sample included 605 patients.

Of the 605 patients, 451 patients received <14 d of antibiotics after their Stage 2 reimplantation surgery (95.6 % of whom received no antibiotics), and 154 patients received at least 14 d of antibiotics after Stage 2 reimplantation, with a median duration of 90 days (interquartile range (IQR) 31 to 276 d). Overall, the two groups were similar (Table 1), with the exception that there were relatively more knee PJI patients in the group that received a prolonged course of antibiotics. As expected in the veteran population, patients were mostly men (94 %), with a mean age of 66 years and an ASA score of 3. The microbiology of the index PJI was likewise similar between the two groups (Table 2). For patients who received ≥14 d of oral antibiotics after Stage 2 reimplantation, the antibiotics used were tetracyclines (48.1 %), oral beta-lactams (27.3 %), and trimethoprim–sulfamethoxazole (18.8 %) (Table 2). For most patients, the antibiotic chosen was active against the organism causing the index infection, but for seven patients (4.5 %) it was inactive and for another eight patients (5.2 %) the activity was not confirmed (lack of susceptibility data for the chosen antibiotic). The most common potential side effects noted in patients who received oral antibiotics were acute kidney injury (9.7 % of patients), nausea (8.4 %), diarrhea (4.5 %), and rash (2.6 %). More than 90 % of patients in both groups had at least 1 year of documented follow-up in the medical record.

**Table 1:**
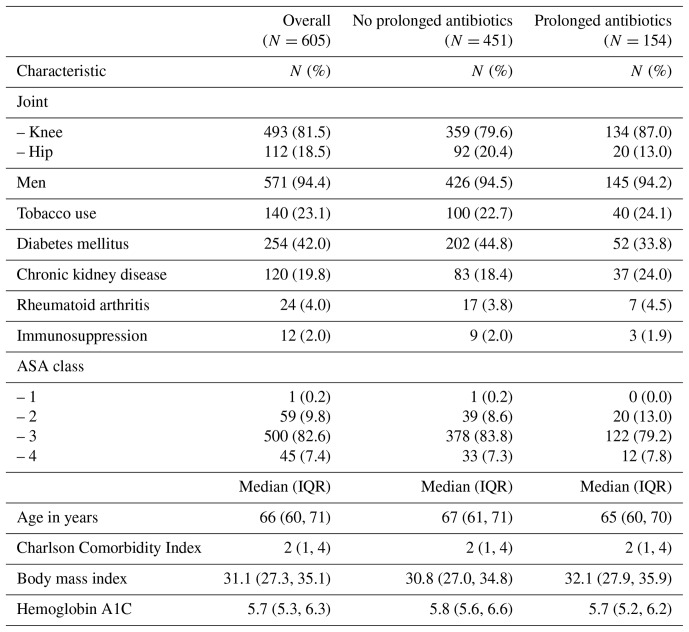
**Table 1**Demographics.

**Table 2:**
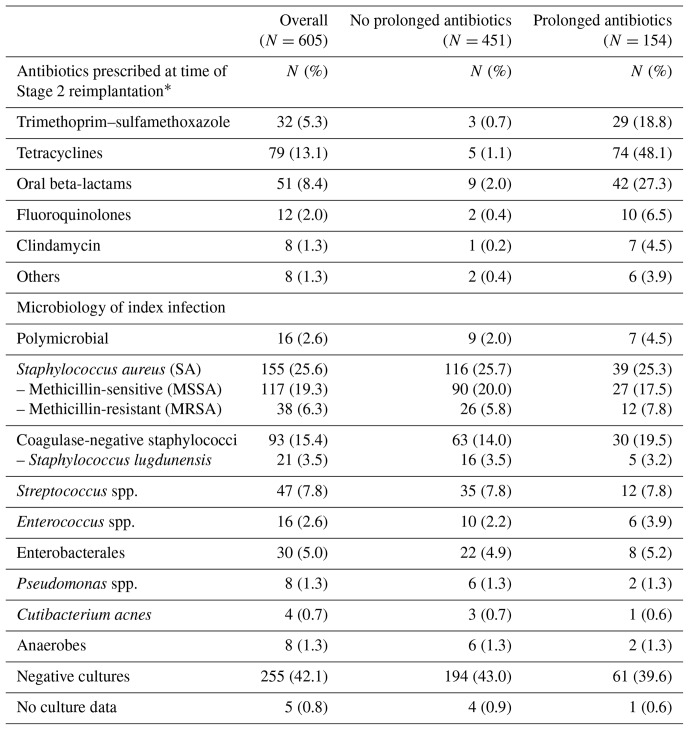
**Table 2**Antibiotics and microbiology.

^*^ Patients could receive more than one antibiotic.

**Figure 1:**
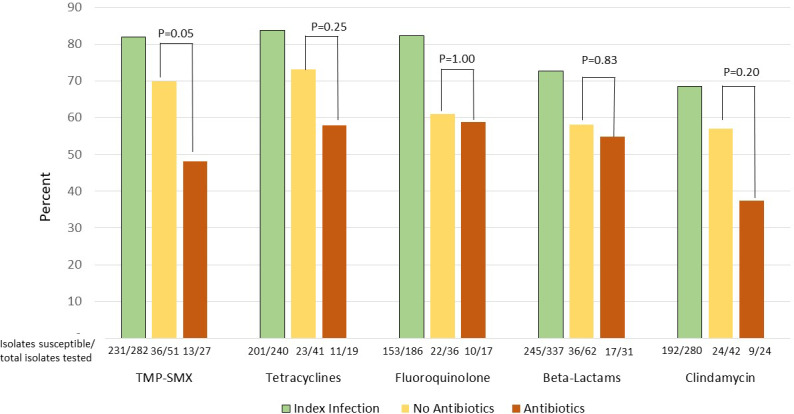
**Figure 1**Percentage of isolates tested against each antibiotic that were susceptible to that antibiotic. For bacterial species with known inherent resistance to a given antibiotic class (e.g., *Pseudomonas* and oral beta-lactams), those isolates were counted as resistant even if not directly tested.

There were 109 patients with recurrent PJI: 80 (17.7 %) in the group that did not receive antibiotics after Stage 2 reimplantation and 29 (18.8 %) in the group that did (Table 3). Eight recurrent PJIs occurred while patients were still on antibiotics; in general, these occurred within 1 to 2 months following the Stage 2 surgery (range 17–78 d post-op). The organisms causing recurrent PJI were similar between the two groups (Table 4). However, patients who had received extended courses of antibiotics appeared more likely to have subsequent infections with organisms resistant to clindamycin, trimethoprim–sulfamethoxazole, and tetracyclines, though this trend only reached statistical significance for trimethoprim–sulfamethoxazole (Fig. 1). Resistance patterns for oral beta-lactams and fluoroquinolones were not appreciably different between the two groups. Analysis of hazard ratios for recurrent PJI and death, adjusted for age, BMI, CCI, and diabetes, showed no difference in risk for recurrence for patients treated with prolonged oral antibiotics compared to those who did not receive antibiotics after Stage 2 reimplantation.

Of the 109 recurrent PJIs, 17 were with the same organism as the index PJI on a species level but only 8 of those had the same resistance profile as the original organism. Seven of the 8 recurrences with apparently the same organism were in patients who had not received extended oral antibiotics after Stage 2 reimplantation. Interestingly, of the nine recurrences with the same organism but with a different resistance profile, five were with a more resistant strain of the same bacteria (four of those five in patients who received extended oral antibiotics) but four were with a *less* resistant strain (all in the group that did not receive extended oral antibiotics).

**Table 3:**
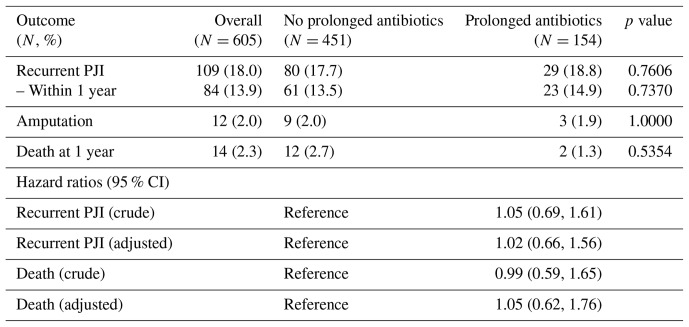
**Table 3**Clinical outcomes.

**Table 4:**
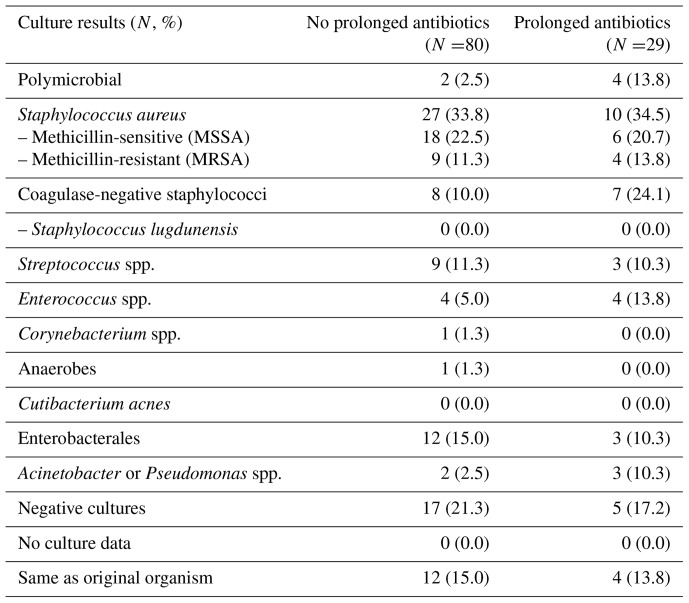
**Table 4**Microbiology of recurrent PJI.

## 4 Discussion

In this national cohort of veterans with a history of hip or knee PJI treated with two-stage exchange, we observed a trend towards increasing antibiotic resistance of organisms causing subsequent PJIs in patients who received a prolonged course of oral antibiotics after Stage 2 reimplantation, compared to those who did not receive antibiotics. While not reaching statistical significance for any antibiotic except trimethoprim–sulfamethoxazole, this finding aligns with findings from our previous work (Kelly et al., 2022) and with studies in other populations (Gafter-Gvili et al., 2012, 2006). Using antibiotics for prophylaxis often works, but this is at the cost of selecting for resistant organisms. It is also notable that there was an increase in the rate of resistant organisms for any recurrent PJI compared to the index PJI – it was just more pronounced in those that received prolonged antibiotics. This shift in microbiology likely reflects the increased exposure to the healthcare system and to antibiotics in general for patients who suffer from PJI.

We did not observe any discernable advantage associated with the use of extended oral antibiotics after Stage 2 reimplantation in this study. However, as a retrospective analysis this study is limited by uncontrolled biases that may have predisposed those patients who received antibiotics to suffer from recurrent PJI. While we tried to adjust our model for potential confounders, it is likely some residual confounding by indication remains. It is also possible that some veterans with recurrent PJI received care for the recurrence outside the VHA system and that this outside care was more likely in the group that did not receive prolonged antibiotics. That said, in our prior study we likewise found no significant difference in recurrent infection risk between those who received prolonged antibiotics after Stage 2 reimplantation and those who did not (Kelly et al., 2022) Given these findings, the protective effect of antibiotics in this setting is likely modest and must be balanced against adverse effects.

The main strength of this study is that it pulls data from a large national dataset, allowing for a robust analysis, but there are limitations. We relied on data pulled from the CDW for many of the outcomes to expedite data collection, which may have missed some data, e.g., cultures from outside of the VHA system. This may explain why the proportion of patients with culture-negative PJI was relatively high in our study (Nelson et al., 2023). However, we manually reviewed a subset of patients, including all patients with recurrent PJI, and determined that the accuracy of CDW-obtained culture data was about 90 %. Finally, despite the large sample size, the occurrence of relatively few cases with recurrent PJI limited our ability to conduct a detailed analysis on which specific bug–drug combinations were particularly prone to selecting for resistance.

Nevertheless, this study has valuable implications for the care of patients with PJI. When patients are put on prolonged courses of antibiotics, they become colonized with bacteria resistant to those antibiotics. Because patients tend to get infected with their own flora, these patients are subsequently more likely to get infected with antibiotic-resistant organisms. Some antibiotics may be worse than others in this respect. A randomized trial of healthy volunteers given trimethoprim–sulfamethoxazole (TMP-SMX), doxycycline, or cephalexin found that patients treated with doxycycline or TMP-SMX subsequently had doxycycline-resistant or TMP-SMX-resistant (respectively) coagulase-negative staphylococci on their skin (Jo et al., 2021) For the patients treated with doxycycline for 2 months, this effect lasted for at least a year in many of the subjects. This study of the skin microbiome corroborates our findings that the effect on resistance patterns for TMP-SMX and tetracyclines appears more pronounced than for beta-lactams.

## 5 Conclusions

In summary, this study reinforces prior findings that administering prolonged courses of antibiotics after Stage 2 reimplantation may increase the risk of infection with antibiotic-resistant organisms in subsequent PJI. Further work is needed to determine the risk vs. benefit associated with specific antibiotic selections and durations of therapy after Stage 2 reimplantation.

## Data Availability

De-identified data are available from the authors upon request.

## Code availability

The statistical analysis code is available from the authors upon request.
